# Impact of a UV-C Multiemitter Disinfection System on Hospital Environmental Bioburden and Inactivation of Clinically Relevant Pathogens

**DOI:** 10.3390/pathogens15030246

**Published:** 2026-02-25

**Authors:** Edgar Fiscal-Baxin, Auria del Carmen López-Hernández, María Fernanda González-Ruiz, Gabriel Carrisoza-Martínez, Adriana Lisbeth Lopez-Avila, Daniela Moreno-Torres, Adolfo López-Ornelas, Clemente Cruz-Cruz, Emilio Mariano Durán-Manuel, Miguel Ángel Loyola-Cruz, Magnolia del Carmen Ramírez-Hernández, Gustavo Esteban Lugo-Zamudio, Oscar Sosa-Hernández, Luis Gustavo Zárate-Sánchez, Paulina Carpinteyro-Espin, Rocio Flores-Paz, Dulce M. Razo Blanco-Hernández, Alicia Jiménez-Alberto, Juan A. Castelán-Vega, Claudia C. Calzada-Mendoza, Juan Manuel Bello-López

**Affiliations:** 1 Hospital Juárez de México, Mexico City 07760, Mexico; 2 Sección de Estudios de Posgrado e Investigación, Escuela Superior de Medicina, Instituto Politécnico Nacional, Mexico City 11340, Mexico; 3 Hospital Nacional Homeopático, Hospitales Federales de Referencia, Mexico City 06800, Mexico; 4 Escuela Nacional de Ciencias Biológicas, Instituto Politécnico Nacional, Mexico City 11340, Mexico

**Keywords:** UV-C disinfection, multiemitter system, hospital critical areas, ESKAPE pathogens, infection control

## Abstract

Healthcare-associated infections remain a central hospital challenge, particularly in critical areas where invasive procedures and microbial contamination overlap. The hospital environment, including air and high-touch surfaces, acts as a persistent microorganism source that favors stability and spread. UV-C disinfection systems have become complementary tools to conventional cleaning. This study evaluated the disinfectant efficacy of a 254 nm multiemitter UV-C system under *in situ* and *in vitro* conditions. A 254 nm UV-C multiemitter system was deployed to eight hospital areas selected for epidemiological relevance. Air and surface sampling were conducted before and after standardized UV-C cycles. The bacterial and fungal aerobiome was quantified (CFU/m^3^) and surfaces were characterized by MALDI-TOF mass spectrometry. *In vitro* assays tested efficacy against planktonic cultures and mature biofilms of clinical ESKAPE isolates and *C. albicans*. The UV-C intervention achieved mean aerobiome reductions above 91.5%, with complete elimination in multiple critical zones. Surface contamination was reduced by 96.1%, including total disinfection across several sampled points. *In vitro* testing showed ≥99.99% to 100% elimination of planktonic microorganisms. Mature biofilms exhibited full loss of viability after UV-C exposure, independent of biofilm architecture and structural complexity. Therefore, the 254 nm UV-C multiemitter system significantly reduced environmental microbial burden in critical hospital areas, supporting its integration within infection-prevention programs and reinforcing environmental biosafety through the control of the microbial sources involved in transmission dynamics.

## 1. Introduction

Healthcare-associated infections (HAIs) represent a problem in hospital institutions where high-complexity procedures are performed, such as mechanical ventilation, use of central venous catheters, urinary catheters, transplants, as well as major surgical interventions that involve cavity opening, among others [1,2,3]. This problem is particularly aggravated when HAIs involve multidrug-resistant pathogens from the ESKAPE group (*Enterococcus faecium*, *Staphylococcus aureus*, *Klebsiella pneumoniae*, *Acinetobacter baumannii*, *Pseudomonas aeruginosa* and *Enterobacter* spp.), which have the ability to evade the action of antimicrobials and establish themselves in hospital environments and form reservoirs on inert surfaces, invasive medical devices in the form of biofilm, among others [4,5,6].

In recent years, yeast-like and filamentous fungi have gained relevance as emerging microbiological threats in hospital environments. In 2022, the World Health Organization (WHO) published its first list of priority fungal pathogens, highlighting *Candida auris*, *C. albicans*, *Aspergillus fumigatus*, and *Cryptococcus neoformans*, since they are recognized as important pathogens due to their ability to generate invasive infections that increase morbidity and mortality rates in immunocompromised patients [7]. These fungal pathogens, in addition to their known virulence and antifungal resistance, show a concerning capacity to persist in the hospital environment, forming reservoirs in water distribution systems and air conditioning systems [6,8]. In particular, *C. auris* has been isolated from medical equipment, beds, floors, and drains, which facilitates its dissemination and the occurrence of hospital outbreaks [8,9].

Thus, fungi are added to ESKAPE pathogens as agents responsible for HAIs, posing challenges for institutional infection control programs. At Hospital Juárez de México (HJM), the evidence generated by our working group has allowed us to show an integral panorama of environmental microbiological contamination and its impact on patient safety. Various studies have documented the presence of bacterial pathogens in hospital scenarios, where clonal dispersion of *A. baumannii* in adult intensive care units (AICU) and its relationship with outbreaks in patients with COVID-19 has been demonstrated [10,11,12], as well as biofilms of ESKAPE bacteria resistant to conventional detergents and disinfectants, a situation that shows the need to improve reprocessing practices of invasive medical devices [13]. Complementarily, we have characterized the hospital aerobiome in transplant rooms, where a seasonal dynamic of potentially pathogenic bacteria was identified, representing a risk for immunocompromised patients [14], as well as fungal colonization in critical COVID-19 patients by various genetically related *Candida* spp. species, indicative of transmission through environmental contamination [15].

Taken together, these findings confirm that the hospital environment represents a relevant reservoir for microorganisms implicated in HAIs, whose persistence is favored by environmental conditions, high healthcare workload, extensive use of medical devices, and limitations in ventilation systems. In this context, ultraviolet type-C (UV-C) disinfection systems have emerged as adjunctive interventions to reduce environmental reservoirs of clinically relevant microorganisms implicated in HAIs, as previously reported under controlled and semi-controlled conditions [16]. However, evidence generated under real-world hospital conditions, particularly in middle-income healthcare settings, remains limited.

Therefore, the aim of this study was to comprehensively evaluate the impact of a UV-C multiemitter disinfection system on airborne and surface-associated environmental bioburden in critical hospital areas, as well as its *in vitro* effect against planktonic microorganisms and mature biofilms of clinically relevant pathogens. In this work, the strengths and limitations of the application of these UV-C radiation technologies as a complementary strategy in hospital infection control programs are analyzed and discussed.

## 2. Materials and Methods

### 2.1. Description of the UV-C Disinfection System

The Helios UV-C system (Surfacide^®^, Waukesha, WI, USA) is an automated environmental disinfection platform based on ultraviolet type C (UV-C) radiation, designed for the elimination of pathogenic and environmental microorganisms on surfaces and air in hospital areas. The system is cleared by the U.S. Food and Drug Administration (FDA) as a Class II medical device. The system is composed of three independent emitting towers, each equipped with low-pressure mercury lamps that emit germicidal radiation at a central wavelength (λ) of 254 nm, considered optimal to induce thymine dimerization of nucleic acids (cyclobutane pyrimidine dimers), inactivating any type of microorganism [17]. The three towers are electronically synchronized through a centralized control system, which allows a uniform distribution of radiation and the reduction in shadowed zones, due to the formation of a parabolic UV-C emission from multiple angles. The system incorporates a three-dimensional laser mapping mechanism, which evaluates the geometric dimensions of the space and automatically calculates the germicidal dose (mJ/m^2^) and the time required to reach the minimum lethal energy. Typical disinfection cycles have an average duration of 20 to 30 min, depending on the size of the area and the spatial configuration of the towers. For the purpose of this study, standardized disinfection cycles of 30 min each were used. The system includes motion and vibration sensors, which automatically stop UV-C emission upon detection of people in the radiation area. Likewise, it has a digital record of the executed cycles, including disinfection time data and irradiation maps, for traceability and quality control.

### 2.2. In Situ Evaluation of the UV-C Multiemitter System (Selection of Hospital Areas)

The study was carried out in eight areas of HJM, selected for their epidemiological relevance, level of complexity in terms of microbiological control, biological risks associated with invasive procedures, and high environmental bioburden. The included areas were: obstetrics and gynecology operating rooms (*n* = 12), central operating room (*n* = 1), bone marrow transplant and recovery rooms (*n* = 4), bone marrow transplant transfer rooms (*n* = 1), pediatric intensive care unit (PICU) cubicles (*n* = 3), AICU cubicles (*n* = 1), staff restrooms of the AICU (*n* = 1), and the Bacteriology laboratory (*n* = 1).

### 2.3. Experimental Strategy and Access to Hospital Areas

To prevent exogenous contamination during the study in the selected critical areas, personnel access was carried out under a standardized biosafety protocol. Previously, surgical handwashing was performed according to the technique recommended by the World Health Organization (WHO), using 2% chlorhexidine surgical soap (PSA, Mexico City, Mexico). Subsequently, sterile personal protective equipment (PPE) was donned, including a sterile surgical cloth uniform, a three-layer face mask, a cap, a hood, and shoe covers. Access to the areas was carried out exclusively through the transfer zone, stepping previously on an adhesive dust-trapping mat to minimize the dragging of particles and microorganisms. Entry of the UV-C multiemitter into the controlled areas was performed after prior cleaning and disinfection of the transport wheels.

### 2.4. In Situ Microbiological Evaluation Pre-Intervention (“Before”)

Prior to the use of the UV-C system, microbiological characterization was performed in each area. Forty surface samples (10 cm^2^) were collected using the swab method, employing Stuart transport medium. The selection of sites considered critical was based on their high contact frequency (surgical tables, handrails, monitors, lamps, sinks, stretchers, biomedical equipment, among others). Samples were enriched in brain heart infusion (BHI) broth at 37 °C for 24–48 h and subcultured on BHI agar for microbiological isolation and identification. In parallel, the bacterial and fungal aerobiome was quantified using the passive sedimentation method with minor modifications [18]. Plates containing culture media were exposed for 1 h at 1.5 m above the floor, distributed at five strategic points according to ISO 14644-1:2015 [19]. Solid media used were BHI agar for aerobic mesophilic bacteria, and potato dextrose agar (PDA) for filamentous fungi supplemented with chloramphenicol (30 μg/mL) and streptomycin (30 μg/mL). After exposure, plates were incubated at 37 °C for 48 h for bacteria and at 28 °C for 72 h for fungi. Colonies were counted and expressed as colony-forming units per cubic meter (CFU/m^3^), applying the Omeliansky equation as follows [14]:

*N* = 5*a* × 10^4^ (*bt*)^−1^,

where N represents the number of CFU/m^3^, *a* the number of colonies per plate, *b* the area of the plate (cm^2^), and t the exposure time (min).

### 2.5. UV-C Disinfection System Intervention (“Disinfection”)

Disinfection was performed in the total absence of personnel, following standardized operational protocols commonly used for hospital room disinfection. Three synchronized emitters were arranged in an equidistant triangular configuration within the area to be disinfected, with the objective of maximizing three-dimensional coverage and minimizing shadowed zones. The central emitter was placed along the longitudinal axis of the area, while the lateral emitters were positioned equidistant from the perimeter walls, oriented toward critical surfaces.

**Figure 1:**
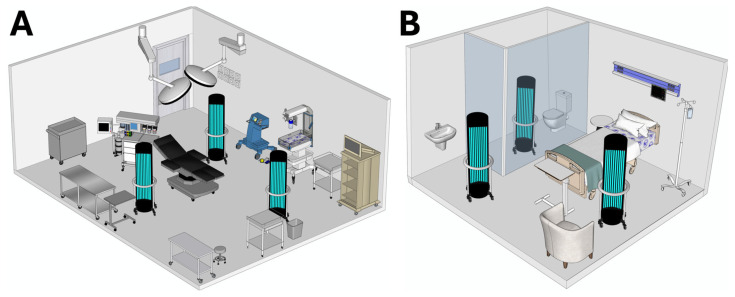
Strategic placement of the multiemitter UV-C towers in hospital environments: (**A**) operating room; (**B**) bone marrow transplant room. These diagrams depict how the towers are positioned to maximize irradiation coverage and minimize shadowed zones created by equipment or furnishings. Schematic diagrams created by the authors using Autodesk AutoCAD 2025.

The irradiation cycle had a standardized duration of 30 min, automatically determined by the integrated dosimetry software, ensuring delivery of an estimated cumulative UV-C dose in the range of approximately 800–1500 mJ/cm^2^ on surfaces within direct line of sight of the emitters. Once the process was completed, the areas were ventilated for 10 min before personnel re-entry. Figure 1 shows two schematic representations of two areas and the strategic positioning of the three UV-C emitters, highlighting their spatial distribution and irradiation angles over the surfaces of interest.

### 2.6. In Situ Microbiological Evaluation Post-Intervention (“After”)

Microbiological measurements were repeated under the same conditions established in the “before” stage, maintaining the same sampling points and collection procedures (for air and surfaces). The microbiological samples obtained were processed and analyzed under identical conditions to those of the “before” phase, to allow a direct quantitative comparison of pre- and post-intervention values.

### 2.7. Disinfectant Efficacy of the UV-C System

The disinfectant efficacy of the UV-C system was measured through the logarithmic and percentage reduction in the bacterial and fungal aerobiome, which was expressed as the logarithmic reduction value (LRV) using the following equation [16]:

*L* = *log*_10_(*A*) − *log*_10_(*B*),

where L is the LRV, A is the number of viable CFU before UV-C disinfection, and B is the number of CFU after UV-C disinfection, expressed as log_10_ (CFU/m^3^). Additionally, Percent Reduction Values (PRV) were calculated using the following equation:

*P* = [(1 − 10^−^*ᴸ*) (100)],

where P is the PRV, and L is the LRV. Since LRV and PRVs were calculated deterministically as efficacy indicators per assay, no additional inferential tests were applied.

### 2.8. In Situ Microbiological Evaluation Post-Intervention (“After”) on Surfaces

To determine the efficacy of the UV-C multiemitter radiation system on hospital surface microbiological contamination, a comparative analysis was performed by culture in LB broth before and after the intervention. Bacterial growth obtained in the “before” phase and in the “after” phase was recorded and transformed into a binary presence/absence matrix for each sampling point, to construct heat maps that visually represented the spatial distribution of microbiological contamination. This approach allowed the identification of sites with the highest initial microbial load and the quantification of reduction after UV-C exposure. Finally, the percentage reduction in contamination was calculated considering the forty surfaces evaluated per procedure and the number of surfaces that maintained residual growth after treatment.

### 2.9. Bacterial Identification by MALDI-TOF Mass Spectrometry

Bacterial isolates obtained in the “before” and “after” stages of UV-C radiation were identified at the genus and species level through direct analysis of whole bacterial cells using matrix-assisted laser desorption/ionization time-of-flight mass spectrometry (MALDI-TOF MS). For this purpose, all strains were streaked on LB agar and incubated overnight at 37 °C. Subsequently, isolated colonies were selected for identification using a Bruker MALDI Biotyper system (Bruker Daltonik, Bremen, Germany), following the manufacturer’s instructions. High-confidence identifications were considered those with score values higher than 2.0 (up to 3.0) according to the identification protocol criteria.

Importantly, all culturable colonies recovered from each surface sample in both the pre-intervention (“before”) and post-intervention (“after”) phases were individually analyzed by MALDI-TOF MS. No sub-sampling strategy was applied; all morphologically distinct colonies obtained after enrichment and subculture were subjected to species-level identification to ensure exhaustive taxonomic characterization of the surface-associated contamination. Genus and species assignment was performed using the MBT Compass Library, version 10.0. With the microbiological data obtained, taxonomic diversity and frequency graphs by evaluated area were generated, comparing pre- and post-intervention results.

### 2.10. In Vitro Evaluation of the UV-C Disinfection System on Planktonic Models

The bacterial and fungal strains used in this study correspond to clinical isolates previously reported (Table 1). Strains were inoculated in Luria–Bertani (LB) broth under agitation at 200 rpm for 24 h at 37 °C for bacteria and 48 h at 28 °C for fungi. Subsequently, cultures were serially diluted in isotonic saline solution to obtain concentrations of 10^1^, 10^2^, 10^3^, 10^4^, 10^5^, 10^6^, and 10^7^ CFU/mL. Microbial suspensions were stored and refrigerated at 4 °C for 18 h before use on an ice bed. Once microbial density was confirmed, suspensions were plated again (in triplicate) on LB agar and exposed to UV-C radiation with the multiemitter system as follows. Exposure of planktonic cultures was performed for 30 min, at 1.5 m and at floor level, strategically placed within a 9 m^2^ enclosure. Controls of planktonic cultures without exposure to UV-C radiation were included. After UV-C irradiation, cultures were incubated under appropriate time and temperature conditions, and surviving CFU were counted. Disinfectant activity of the UV-C radiation multiemitters was determined by calculating LRV and PRVs, considering viable CFU/mL of control cultures and CFU/mL of irradiated cultures.

### 2.11. In Vitro Evaluation of the UV-C Disinfection System on Mature Biofilms

Microbial strains were cultured in 3 mL of LB broth under appropriate incubation conditions of temperature and time. Microbial cultures were centrifuged, cold isotonic saline solution was added to the microbial pellet, and suspensions were adjusted to 0.5 McFarland nephelometer standard. Bacterial and fungal suspensions (50 μL) were inoculated in triplicate in BHI broth (3 mL) into wells of a flat-bottom plate (Corning, Kennebunk, ME, USA). Plates were sealed and incubated at 37 °C for 48 h (bacteria) and 28 °C for 72 h (fungi). Non-adherent cells (planktonic) were removed from the plate by aspiration. Well plates were gently washed with 1× PBS (pH 7.4) and subjected to UV-C light treatment according to Section 2.5. Biofilms were stained using fluorescent dyes to identify live and dead bacteria and fungi by fluorescence microscopy. Syto9/PI-stained biofilms allowed monitoring of viability as a function of membrane integrity. Cells with compromised membranes were red, whereas cells with intact membranes were green. Finally, biofilms were fixed using 4% paraformaldehyde and washed with sterile 1× PBS. Images were acquired on a Cytation 5 Cell Imaging Multimode Reader (Agilent, Santa Clara, CA, USA). Viable and non-viable cells were observed at 488 nm and 543 nm, respectively, and counted using Gen5 software. Colony-forming units’ enumeration from biofilms (after mechanical/enzymatic detachment and plating) was not performed, as the study endpoint for mature biofilms was fluorescence-based loss of viability.

## 3. Results

### 3.1. Baseline Characteristics of the Aerobiome “Before” UV-C Disinfection

To establish a quantitative panorama of the bacterial aerobiome in the evaluated areas, the CFU/m^3^ concentrations obtained before the UV-C intervention were classified into three bioburden levels (low, medium, and high), based on the tertiles of the distribution of recorded values. The determined limits were ≤13.1 CFU/m^3^ for low load, from 13.2 to 21.0 CFU/m^3^ for medium load, and >21.0 CFU/m^3^ for high load. Of the 24 areas analyzed, ten (41.7%) presented low bacterial load, seven (29.2%) showed medium levels, and seven (29.2%) exhibited high concentrations. The areas with the highest bacterial bioburden corresponded to the AICU cubicles (F), the PICU (E), and the Bacteriology laboratory (H). In these zones, values ranged from 104.8 to 183.4 CFU/m^3^. In contrast, areas with lower bacterial load mainly included operating rooms and controlled areas (A, B, and C). The bioburden values of the evaluated hospital areas are shown in Table 2.

Analogously to the bacterial analysis, fungal aerobiome concentrations before exposure to UV-C radiation were classified into the same three levels based on distribution tertiles. Values ≤10.5 CFU/m^3^ were considered low load, those from 10.6 to 16.9 CFU/m^3^ were medium load, and those >16.9 CFU/m^3^ were high load. In total, nine areas (37.5%) showed low fungal concentration, seven (29.2%) presented medium levels, and eight (33.3%) showed high concentrations.

Zones with the highest spore density mainly corresponded to the Bacteriology laboratory (H), PICU cubicles (E), obstetrics and gynecology operating room (A), AICU restroom (G), bone marrow transplant area (D), and post-transplant recovery rooms (C). In these areas, concentrations ranged from 19.6 to 85.1 CFU/m^3^. In contrast, spaces classified with low load (5.2 to 10.5 CFU/m^3^) included some operating rooms (A and B) (Table 1). This classification of the bacterial and fungal aerobiome served as a reference point to evaluate efficacy after the intervention. Bioburden results showed that environmental contamination was dominated by bacteria rather than fungi.

### 3.2. In Situ Evaluation of the UV-C Disinfection System (Bacterial Aerobiome) “After”

After intervention with the UV-C multiemitter system, an average PRV of 91.5% was observed in bacterial bioburden across the twenty-four hospital areas evaluated. Post-intervention bioburden values ranged from 0.0 to 70.7 CFU/m^3^, showing a homogeneous pattern of bioburden decrease after exposure to UV-C radiation. Obstetrics and gynecology operating rooms and the general operating room (A and B), the bone marrow transplant and recovery room (C), as well as PICU cubicles and the AICU restroom (E and G), achieved complete bioburden reductions (100%). Other evaluated areas showed reductions ranging from 35.4% to 96.9%. Interestingly, areas with the highest baseline bioburden (E, F, and H) were those that showed lower PRVs (Table 2).

### 3.3. In Situ Evaluation of the UV-C Disinfection System (Fungal Aerobiome) “Before”

After intervention with the UV-C multiemitter system, the fungal aerobiome showed an average PRV of 93.4% across the twenty-four hospital areas evaluated. This decrease was consistent in most areas. Residual post-intervention bioburden ranged from 0.0 to 5.2 CFU/m^3^, evidencing a substantial reduction in fungal bioburden. The highest efficiencies were recorded in obstetrics and gynecology operating rooms (A), the bone marrow transplant room (C), as well as adult and PICU cubicles and staff restrooms of the AICU (E, F, and G), where PRVs of 100% were achieved. In contrast, partial reductions were observed, particularly in some obstetrics and gynecology operating rooms and the general operating room (A and B), with decreases ranging from 33.3% to 85.7% (Table 2).

### 3.4. In Situ Evaluation of the UV-C Disinfection System (Surfaces)

**Figure 2:**
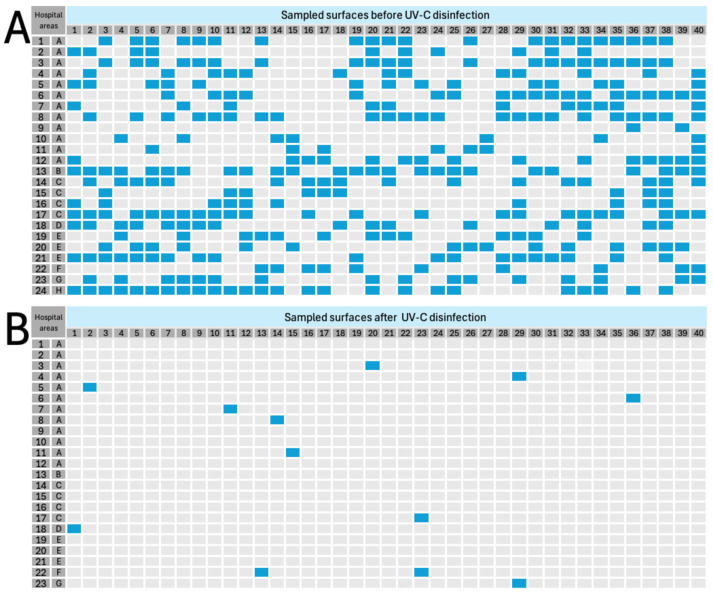
Evaluation of surfaces’ contamination before (**A**) and after (**B**) in UV-C multiemitter disinfection assays. Blue squares represent surfaces where microbial contamination was detected, while gray squares correspond to surfaces without microbial growth. Hospital areas: obstetrics and gynecology operating rooms (1A – 2A), central operating room (13B), bone marrow transplant and recovery rooms (14C – 17C), bone marrow transplant transfer rooms (18D), pediatric intensive care unit (PICU) cubicles (19E – 21E), AICU cubicles (22F), staff restrooms of the AICU (23G), and the Bacteriology laboratory (24H).

Figure 2 shows the spatial distribution of bacterial contamination on 960 high-touch surfaces (40 per area) in the 24 hospital areas evaluated, before (panel A) and after (panel B) the intervention with the UV-C multiemitter radiation system. In the “before” phase, 361 surfaces (37.6%) were positive for bacterial growth, with heterogeneous dispersion patterns among areas (Figure 2A). The highest contamination rates were located in obstetrics and gynecology operating rooms (A) and the general operating room (B), bone marrow transplant and recovery rooms (C), PICU cubicles (E), and the Bacteriology laboratory (H), where the proportion of contaminated surfaces ranged from 48% to 63%, coinciding with the highest baseline airborne bioburden values recorded in these zones (Table 2). After UV-C radiation intervention (“after” panel), a generalized transition from positive to negative cultures was observed, reflecting a marked and consistent decrease in bacterial contamination. Only 13 surfaces remained positive, representing a 96.1% reduction compared to initially contaminated surfaces (*n* = 361). In addition, no culturable microorganisms were detected on multiple surfaces after UV-C intervention, particularly in operating rooms (A), bone marrow transplant rooms (C), and PICU cubicles (E). In hospital areas that showed residual contamination, disinfection efficiency ranged from 80% to 95.65% (Figure 2B).

### 3.5. Decrease in Taxonomic Diversity After UV-C Surface Disinfection

**Figure 3:**
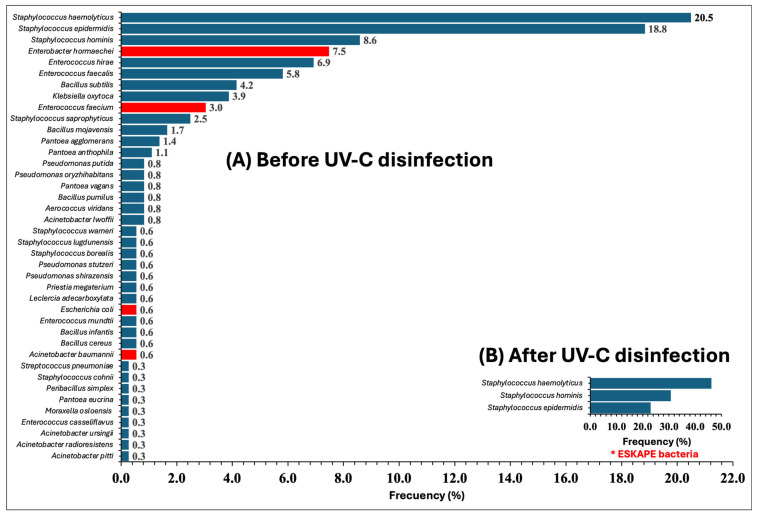
Frequency of bacteria isolated from surfaces before (**A**) and after (**B**) UV–C multiemitter disinfection assays. Bars represent the frequency of each identified species. In graph (**A**), high bacterial diversity is observed, including species from the ESKAPE group (*), while in (**B**), a reduction in frequency and microbial diversity after UV–C disinfection is observed.

Figure 3 shows the bacteriological characterization of surface contamination by MALDI-TOF mass spectrometry in the 24 evaluated areas of HJM, comparing the “Before UV-C disinfection” and “After UV-C disinfection” stages. Results revealed that of the 960 surfaces subjected to culture, only 361 surfaces showed initial contamination, and after UV-C intervention, only 13 isolates were recovered. Diversity analysis showed that in the initial stage (before), 40 different taxa were identified, consisting of Gram-negative and Gram-positive bacteria, with *S. haemolyticus*, *S. epidermidis*, and *S. hominis* being the most abundant, with identification frequencies of 20.5%, 18.8%, and 8.6%, respectively. In bacteriological terms, the baseline condition of the analyzed areas (before intervention) reflected contamination with high microbial heterogeneity, with environmental and commensal bacteria being the most frequent (blue bars).

Genera and species such as *P. agglomerans*, *P. anthophila*, *P. putida*, *B. subtilis*, *B. mojavensis*, and *P. megaterium* were the most abundant as environmental contaminants. After UV-C disinfection, a significant 92.5% reduction in taxa (*n* = 37) was detected, with recovery of only three species of the genus *Staphylococcus* (*n* = 7.5%), such as *S. haemolyticus*, *S. hominis*, and *S. epidermidis*, which were those that showed the highest isolation frequency in the “before” stage. Complementarily, when evaluating bacteriological variation between both stages, it was observed that in the “before” stage, the microbial profile was largely distributed among multiple genera and species of bacteria from the ESKAPE group (*n* = 10), representing 25% of the total detected taxa. The detected ESKAPE genera and species were *E. hormaechei*, *K. oxytoca*, *E. faecium*, *P. agglomerans*, *P. anthophilia*, *L. adecarboxilata*, *E. coli*, *A. baumannii*, and *P. eucrina*, with most of these being members of the *Enterobacterales* order (Figure 3).

### 3.6. In Vitro Evaluation of the UV-C Disinfection System on Planktonic Models

Table 3 shows the Percent Reduction Values (PRV) obtained after exposure of planktonic cultures of ESKAPE group bacteria and *C. albicans* to the UV-C multiemitter radiation system. All strains used correspond to clinical isolates obtained at HJM, previously characterized as highly virulent, biofilm-forming, and multidrug-resistant to antibiotics. In planktonic cultures with initial concentrations of 10^7^ and 10^6^ CFU, a ≥99.99% reduction was observed in all analyzed species. In the case of *K. pneumoniae*, *E. coli*, and *C. albicans*, a PRV of 99.99% was observed at densities of 10^5^ CFU, with the latter being more resistant to UV-C irradiation, since at concentrations of 10^4^ and 10^3^ CFU a PRV of 99.99% was also observed. Finally, all employed models showed PRVs of 100% at densities of 10^2^ and 10^1^ CFU.

### 3.7. In Vitro Evaluation of the UV-C Disinfection System on Mature Biofilms

**Figure 4:**
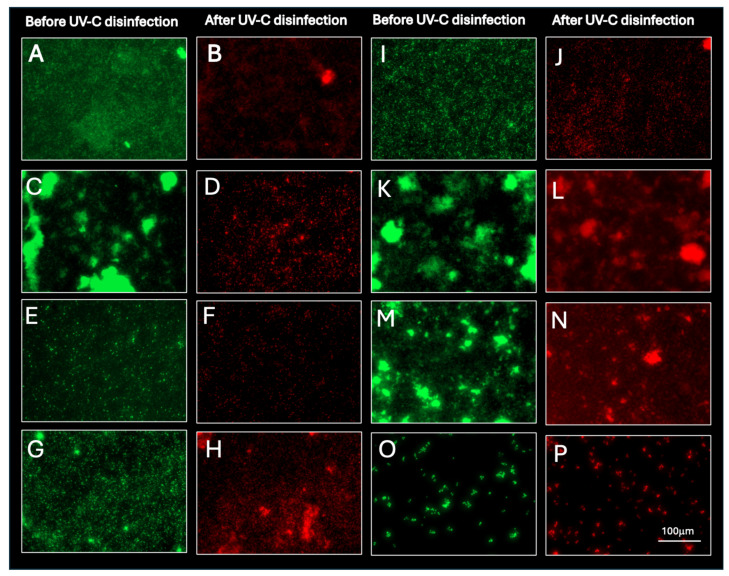
Viability of mature biofilms before and after disinfection with the UV-C multiemitter. Green panel (before disinfection) and red panel (after disinfection). (**A**,**B**): *Acinetobacter baumannii*; (**C**,**D**): *Staphylococcus aureus*; (**E**,**F**): *Pseudomonas aeruginosa*; (**G**,**H**): *Citrobacter freundii*; (**I**,**J**): *Klebsiella pneumoniae*; (**K**,**L**): *Escherichia coli*; (**M**,**N**): *Stenotrophomonas maltophilia*; (**O**,**P**): *Candida albicans*. Scale bar: 100 μm.

Figure 4 shows the viability of mature biofilms before and after exposure to the UV-C multiemitter system. Before exposure to UV-C radiation, green fluorescence predominated, indicative of a viable biofilm. Differences in fluorescence intensity depended on the microbial species. *Acinetobacter baumannii* (A), *S. aureus* (C), *E. coli* (K), and *S. maltophilia* (M) formed biofilms with dense and compact clusters. In contrast, strains such as *P. aeruginosa* (E), *C. freundii* (G), *K. pneumoniae* (I), and *C. albicans* (O) formed biofilms with more uniform and less complex distribution. After UV-C irradiation, red fluorescence (indicative of microbial death) was predominantly observed in all microorganisms, although the fluorescence pattern again depended on the characteristics of each biofilm. In both types of biofilms (uniform and dense), disappearance of the viability signal was complete, indicating total inactivation (B, D, F, H, J, L, N, and P).

## 4. Discussion

Healthcare-associated infections are one of the most important challenges in hospital centers, and it is here where infection control strategies play a central role in their containment. Because HAIs are multifactorial, disinfection technologies are added as measures that, together with already known strategies such as adherence to hand hygiene, cleaning procedures, and isolation of patients with HAIs, among others, have demonstrated their effectiveness [21]. Disinfection technologies have advantages due to their ability to eliminate a wide diversity of environmental microorganisms and pathogens; however, in recent years, systematic reviews have shown that short-wavelength UV-based disinfection shows important advantages in several aspects, including the range of microorganisms that can be annihilated, ease of use, and costs, among others [22]. Although UV-C light has been mostly effective, its use in hospitals in developing countries remains limited due to high initial costs and technical requirements for its implementation. Thus, hospital centers in Latin America maintain traditional manual disinfection methods, which, although less effective, are accessible and easy to implement. Various studies agree that cost and operational complexity are the main barriers to incorporating UV-C robots in resource-limited environments [23,24]. In this context, at the international level, several studies have been published evaluating the efficacy of robotic UV-C disinfection systems in clinical environments, mainly in Europe, North America, and Asia [25,26,27]. However, to our knowledge, in Latin America, there are no studies documenting in situ disinfectant evaluation of this type of technology in hospitals in the region, nor under controlled laboratory conditions (in vitro) against complex microorganisms such as ESKAPE bacteria and biofilms. Therefore, the absence of local evidence allowed evaluation of this system in a third-level Mexican hospital, Hospital Juárez de México.

The results presented in Table 2 confirm the high efficacy of the UV-C multiemitter radiation system to reduce environmental microbial bioburden in the evaluated areas. The average PRV reduction was greater than 90% for the bacterial and fungal aerobiome, which highlights that UV-C radiation is a robust disinfection tool, without ceasing to be considered as a complementary method to conventional infection control strategies. An important point to note is that although germicidal UV-C radiation has been evaluated in numerous studies for reducing microorganisms on hospital surfaces, no literature has been identified on its disinfectant effect on bacterial and fungal aerobiome in real hospital environments using technologies originally oriented toward surfaces. The few studies on UV-C air disinfection are usually under specific experimental conditions, without considering real hospital scenarios [28]. Therefore, we consider that the present work is the first report demonstrating that a UV-C disinfection technology designed for surfaces can significantly reduce aerobiome load in a real clinical context.

As observed in Table 2, in most disinfection assays, UV-C radiation reduced microorganism populations to non-detectable levels; in some cases, the reduction was not as effective as expected. It must be recognized that some cases were identified where reduction ranged from 35.4% to 33.3% for bacteria and fungi, respectively. It must be recognized that, although radiation was three-dimensional, there were possibly zones where microbiological contamination could not be eliminated. We speculate that this variability observed among evaluated hospital areas reflects the influence of architecture, airflow, and density of biomedical equipment, among others. For example, intensive care units (including restrooms) and the microbiological diagnostic laboratory, with greater structural complexity and equipment density, showed lower PRVs compared to operating rooms or transplant rooms (Table 2). Improvements such as increasing dose/time could be the solution so that in those zones that presented lower PRVs, similar or equal values to 100% could be achieved. Previous works highlight the importance of angular distribution and effective dose in the germicidal efficiency of UV-C radiation, which could confirm our hypotheses [29,30]. Future work will be directed to determine the efficiency of this multiemitter system using UV-C radiation exposure time as a variable and its impact on the reduction in bacterial and fungal aerobiome. Now, it is well known that these technologies are primarily designed for surface disinfection, which is why a parallel experiment to aerobiome reduction was designed, dedicated to the disinfection of critical high-touch surfaces. As can be observed in Figure 2A, the high frequency of contaminated surfaces before irradiation in the 24 evaluated areas (*n* = 361 of 960) highlights two critical situations.

First, the presence of contamination in hospital areas where invasive medical procedures are performed, such as mechanical intubation, major surgeries, and catheter insertion, among others, and second, this contamination was detected when the areas had previously been subjected to cleaning and disinfection processes (nebulized hydrogen peroxide). In this type of scenario, the presence of residual contamination after cleaning and disinfection indicates a potential risk of cross-contamination between surfaces, healthcare personnel, and patients. These types of microbiological situations can generate important events such as clonal dispersion of medically relevant bacteria (for example, *A. baumannii*), and recontamination of surfaces after cleaning and disinfection with standard methods, even when disinfectants such as isopropyl alcohol, chlorine-releasing agents, or sodium hypochlorite have been used [10,31]. On the other hand, identification of bacterial contamination on surfaces, although revealing a wide range of environmental and commensal contaminants, many of these have been involved in HAIs, such as *S. haemolyticus*, which has been implicated in bacteremias, especially in the presence of medical devices [32]. Likewise, *S. epidermidis* is a common cause of infections related to catheters and other invasive devices [33], and *E. hirae* has been reported in cases of bacteremia and other hospital infections [34].

Similarly, although species of the genus *Bacillus* have traditionally been considered strictly environmental microorganisms, in recent years several studies have documented their role as opportunistic pathogens involved in HAIs [35,36,37]. Thus, the results shown in Figure 3 not only reflect environmental and commensal microbiological diversity but also the latent risk of these microorganisms becoming potential opportunistic pathogens. Another important aspect was that among microorganisms detected on surfaces, several isolates corresponded to pathogens from the ESKAPE group, already detected as contaminants and generators of HAIs at HJM. For example, species of the genus *Enterobacter*, including *E. hormaechei*, have been described as causative agents of ventilator-associated pneumonia (VAP) and as contaminants in hematopoietic progenitor cell areas [11,14]. Although *K. oxytoca* has not been recognized as a potential HAI generator in our hospital and is not formally included within the ESKAPE acronym, its identification at 3.9% shows that risk is latent due to its presence as a contaminant, since, according to previous works, it has been recognized as an intrahospital pathogen and identified as an environmental contaminant [38,39]. Another member detected with important frequency (3.0%) was *E. faecium*, a Gram-positive ESKAPE member recognized for its medical importance, especially when showing vancomycin resistance, due to its role in causing multiple infections and its identification as a hospital contaminant [40].

The genus *Pantoea*, being an enterobacterium, places all its species within the *Enterobacterales* order; therefore, the presence of *P. agglomerans* (1.4%), *P. anthophila* (1.1%), *P. vagans* (1.4%), and *P. eucrina* (0.3%) constitutes a focus of attention, particularly *P. eucrina*, which has been identified as an important contaminant on healthcare personnel gowns and surfaces in our hospital [10,41]. *Leclercia adecarboxylata* and *E. coli*, although less frequent in isolation, have been implicated in diverse infections, with the COVID-19 pandemic being the period when *E. coli* played an important role as an HAI generator [12,42]. In the clinical context of HJM, *A. baumannii* has been an ESKAPE member with an important role due to its persistence as part of the microbiological reservoir and the identification of MDR strains and high-risk sequence types [10,12].

Its identification, even at a low frequency (0.6%), implies a risk due to cross-contamination events that could give rise to HAIs (Figure 3). The description of the microbiological context of the “(A) before disinfection” stage shows the impact that UV-C radiation had on evaluated surfaces, since the reduction in contamination frequency by 96.4% supports the capacity of the UV-C system to annihilate surface contamination to levels so low that only three environmental and commensal bacterial genera were detected (*S. haemolyticus*, *S. hominis*, and *S. epidermidis*), as shown in the “after UV-C disinfection” panel B. The importance of surface disinfection with broad-spectrum technologies lies in the fact that various authors have reported the persistence of microorganisms on monitors, handrails, and medical equipment even after exhaustive manual cleaning [6,10,43]. In addition to its broad spectrum of contaminant elimination, UV-C radiation offers additional advantages such as not generating chemical residues, not damaging surfaces (as hydrogen peroxide does), reducing sanitization times, and being cost-effective compared to other methods such as peroxide nebulization [43].

In situ results showed average disinfection efficiencies above 90%, which could seem contradictory to what many companies that manufacture these technologies indicate, since they report elimination of 99.99% of microbiological contamination. Under this background, it is important to note that there are multiple variables that may be involved and could influence the level of disinfection without reaching 99.99%. For this reason, disinfection assays were performed under laboratory conditions. For this purpose, we decided to control bacterial density as a variable in disinfection assays, including the absence of any shadow formation, as well as delimiting microbial density to a defined flat surface of 28 cm^2^, equivalent to the area of a conventional culture Petri dish (see Materials and Methods). The results of the in vitro assays confirmed the effectiveness of the system against bacteria of medical interest with phenotypic and genotypic characteristics of virulence and antimicrobial resistance (Table 3). Genera and species from the ESKAPE group, including a yeast-like fungus of medical importance (*C. albicans*), were successfully inactivated after UV-C exposure, which is consistent with previous findings by our research group using portable UV-C irradiators [16].

As shown in Table 3, when bacterial concentration (in base-10 logarithm) is controlled on a total surface of 28 cm^2^, PRVs of 99.99% are achieved even when 10^7^ total CFU are subjected to disinfection, that is, ten times more than what we have reported in previous works on bacterial disinfection using UV-C [16]. Only *C. albicans* showed slight resistance at several lower concentrations (10^3^ to 10^7^ CFU); we speculate that this slight resistance is attributable to the greater complexity of its cell wall, DNA repair capacity, production of melanin pigments, and ability for morphological transition [44]. With this evidence, it becomes clear that the disinfectant capacity of this system reaches 99.99% when the variables described above are controlled. This experimental approach allowed control of some variables; however, it must be recognized that the exclusive use of microorganisms in a planktonic state represents a limitation of the present study, compared with models that evaluate the efficacy of UV-C multiemitter systems on real surfaces and diverse materials within hospital rooms (surface tests) [45].

Nevertheless, this experimental design was intentional and allowed direct evaluation of the disinfectant effect of UV-C radiation on microbial viability, reducing interference from additional physical factors such as materials and surfaces. It is important to indicate that planktonic assays are fundamental to establishing germicidal efficacy under controlled conditions; in fact, a large part of disinfection assays is carried out with microbial models in this form. However, these do not show a real panorama of microbial resistance structures, such as biofilms. For this reason, disinfection tests against mature biofilms of the ESKAPE group and *C. albicans* were developed to approximate one of the ways in which pathogens are found in critical hospital areas. The results of these assays clearly showed the disinfectant power of UV-C radiation even when microorganisms were organized in complex structures with the presence of molecules involved in microbial aggregation and adhesion, such as polysaccharides, proteins, and other biomolecules [46].

An additional finding, which could be considered a limitation, was the inability of UV-C radiation to disrupt biofilms, since only cell death was observed without an apparent change in biofilm complexity (Figure 4). In contrast, other works have reported significant disruption of biofilms on wet and dry surfaces; however, such conclusions are mainly based on quantification assays using crystal violet, a technique that evaluates total adhered biomass and does not discriminate between viable cells, non-viable cells, and extracellular matrix [47]. In this sense, the apparent discordance between the two results can be explained by differences in the methodologies employed. While crystal violet reflects changes in the amount of adhered biomass, confocal microscopy allows direct evaluation of cell viability and structural integrity of the biofilm. Thus, UV-C radiation induced microbial death without causing disruption, a phenomenon that could be interpreted differently depending on the method used. A limitation is that biofilm inactivation was assessed by LIVE/DEAD fluorescence viability imaging rather than CFU recovery after biofilm disruption; future studies could complement these findings with standardized detachment and CFU-based quantification. Finally, it is important to consider that in commercial UV-C disinfection systems designed for use in real hospital environments, efficacy is not usually expressed as an accumulated physical dose (J/m^2^), but rather as the result of exposure cycles previously validated as a function of time. For this reason, in the present work, standardized exposure times of 30 min of UV-C were considered, corresponding to the operational cycles commonly used for complete room disinfection. This approach, closer to everyday clinical practice, differs from that used in controlled experimental studies, where dose is directly quantified. This study did not assess healthcare-associated infection rates or patient outcomes; therefore, the observed microbiological reductions should be interpreted as surrogate indicators of environmental risk reduction rather than direct clinical impact.

## 5. Conclusions

The *in situ* reduction in airborne microbial load above 91.5%, together with the elimination of microbiological contamination on surfaces above 95%, as well as the inactivation of microorganisms in planktonic state at 99.99% and the total loss of viability in mature biofilms, support the integration of UV-C multiemitter systems as a complementary component of infection prevention strategies in critical hospital areas.

## Figures and Tables

**Table 1:** Strains used in this study.

Strains	Source	Phenotype and/or Genotype *	Reference
*Acinetobacter baumannii*	Lung	Strong biofilm former, MDR, *bla_VIM_*, *AdeABC*	[10]
*Staphylococcus aureus*	Lung	Strong biofilm former, *mecA*, *SSCmec* IIb	[10]
*Pseudomonas aeruginosa*	Lung	Strong biofilm former, MDR, *bla_VIM_*,	[11]
*Citrobacter freundii*	Blood	Strong biofilm former, IMI^R^, ET^R^	[11]
*Klebsiella pneumoniae*	Wound	Strong biofilm former, *bla_OXA-48_*	[12]
*Escherichia coli*	Urine	Strong biofilm former, *bla_NDM-1_*	[12]
*Stenotrophomonas maltophilia*	Blood	Strong biofilm former, ST92, *fhaB*, *afaD fimH*, *pilU*	[20]
*Candida albicans*	Lung	Strong biofilm former, *ALS1*, *INT1*, *HWP1*	[15]

* MDR: Multidrugresistant; *bla_VIM_* and *bla_NDM-1_*: Metallo b-lactamase genes; *bla_OXA-48_*: serine b-lactamase gene *AdeABC*: efflux pump operon; *mecA:* methicillin resistance gene, *SSCmec* IIb: Staphylococcal chromosomal cassette mec; IMI^R^: imipenem resistance; ET^R^: ertapenem resistance; *fhaB*: filamentous hemagglutinin adhesin gene; *afaD*: afimbrial adhesin regulation/assembly protein gene; *fimH*: type 1 fimbrial adhesin gene; *pilU*: type IV pili twitching motility protein gene; *ALS1:* agglutinin-like sequence 1 adhesin gene; *HWP1*: hyphal wall protein 1 adhesin gene; *INT1*: integrin-like protein 1 adherence gene.

**Table 2:** Bacterial and fungal aerobiome quantification (CFU/m^3^) before and after UV-C disinfection and Percent Reduction Values (PRV) in different hospital areas.

Disinfection Assay	Area	Bacterial Aerobiome			Fungal Aerobiome		
		CFU/m^3^		Percent Reduction Value	CFU/m^3^		Percent Reduction Value
		Before	After		Before	After	
1	A	7.9	0.0	100	29.5	2.6	92.7
2	A	5.2	0.0	100	7.9	0.0	100
3	A	18.3	2.6	85.7	13.1	0.0	100
4	A	18.3	5.2	71.4	6.5	0.0	100
5	A	21.0	0.0	100	5.2	0.0	100
6	A	29.5	2.6	96.9	7.9	0.0	100
7	A	13.1	0.0	100	10.5	0.0	100
8	A	15.7	0.0	100	7.9	5.2	33.3
9	A	15.7	0.0	100	5.2	0.0	100
10	A	13.1	2.6	80	10.5	0.0	100
11	A	15.7	0.0	100	13.1	2.6	80
12	A	10.5	0.0	100	13.1	2.6	85.7
13	B	2.6	0.0	100	15.7	2.6	83.3
14	C	21.0	5.2	75.0	19.6	0.0	100
15	C	13.1	0.0	100	28.8	5.2	81.8
16	C	13.1	0.0	100	13.1	0.0	100
17	C	49.8	5.2	89.5	15.7	0.0	100
18	D	28.8	2.6	90.9	34.1	2.6	92.3
19	E	150.6	13.1	90	32.7	5.2	99.4
20	E	5.2	0.0	100	5.2	0.0	100
21	E	31.4	2.6	91.5	39.3	0.0	100
22	F	183.4	15.7	89.3	16.4	0.0	100
23	G	5.2	0.0	100	42.6	0.0	100
24	H	104.8	70.7	35.4	85.1	5.2	92.6

*Hospital areas:* obstetrics and gynecology operating rooms (1A – 2A), central operating room (13B), bone marrow transplant and recovery rooms (14C – 17C), bone marrow transplant transfer rooms (18D), pediatric intensive care unit (PICU) cubicles (19E – 21E), AICU cubicles (22F), staff restrooms of the AICU (23G), and the Bacteriology laboratory (24H).

**Table 3:** Percent reduction values (PRV) of microorganisms (ESKAPE bacteria and *Candida albicans*) after UV-C disinfection in planktonic form.

Strain Tested	PRV (%)/Microbial Density Tested in Planktonic Form (CFU)						
	10^7^	10^6^	10^5^	10^4^	10^3^	10^2^	10^1^
*Acinetobacter baumannii*	99.99	99.99	100	100	100	100	100
*Pseudomonas aeruginosa*	99.99	99.99	100	100	100	100	100
*Klebsiella pneumoniae*	99.99	99.99	99.99	100	100	100	100
*Citrobacter freundii*	99.99	99.99	100	100	100	100	100
*Escherichia coli*	99.99	99.99	99.99	100	100	100	100
*Stenotrophomonas maltophilia*	99.99	99.99	100	100	100	100	100
*Candida albicans*	99.99	99.99	99.99	99.99	99.99	100	100

## Data Availability

The data presented in this study are openly available in [Mendeley Data] at [https://doi.org/10.17632/c8mw46jf3z.1], reference number [48].

## References

[1] Guarente L., Savinelli S., Masia M., D’Amore G., Di Domenico E. (2024). Device-associated healthcare-associated infection (DA-HAI): Risk factors and preventive strategies. Int. J. Infect. Dis..

[2] Liu X., Chen Y., Wang J., Zhang P., Li H. (2023). Risk factors for healthcare-associated infections: A systematic review and meta-analysis. J. Hosp. Infect..

[3] Czerniak P., Wróblewska M., Piwowar M. (2024). Risk factors for healthcare-associated infections: A single ward study. Med. Res. J..

[4] Mulani M.S., Kamble E.E., Kumkar S.N., Tawre M.S., Pardesi K.R. (2019). Emerging strategies to combat ESKAPE pathogens in the era of antimicrobial resistance: A review. Front. Microbiol..

[5] De Oliveira D.M.P., Forde B.M., Kidd T.J., Harris P.N.A., Schembri M.A., Beatson S.A., Paterson D.L., Walker M.J. (2020). Antimicrobial resistance in ESKAPE pathogens. Clin. Microbiol. Rev..

[6] Osman A.H., Darkwah S., Kotey F.C.N., Odoom A., Hotor P., Dayie N.T.K.D., Donkor E.S. (2024). Reservoirs of nosocomial pathogens in intensive care units: A systematic review. Environ. Health Insights.

[7] World Health Organization (2022). WHO Fungal Priority Pathogens List to Guide Research, Development and Public Health Action.

[8] Silva I., Miranda I.M., Costa-de-Oliveira S. (2024). Potential environmental reservoirs of *Candida auris*: A systematic review. J. Fungi.

[9] Spagnolo A.M. (2025). *Aspergillus* contamination in healthcare facilities: An ever-present issue—Prevention and control measures. Hygiene.

[10] Durán-Manuel E.M., Cruz-Cruz C., Ibáñez-Cervantes G., Bravata-Alcantará J.C., Sosa-Hernández O., Delgado-Balbuena L., León-García G., Cortés-Ortíz I.A., Cureño-Díaz M.A., Castro-Escarpulli G. (2021). Clonal dispersion of *Acinetobacter baumannii* in an intensive care unit designed to patients COVID-19. J. Infect. Dev. Ctries..

[11] Loyola-Cruz M.Á., Durán-Manuel E.M., Cruz-Cruz C., Márquez-Valdelamar L.M., Bravata-Alcántara J.C., Cortés-Ortíz I.A., Cureño-Díaz M.A., Ibáñez-Cervantes G., Fernández-Sánchez V., Castro-Escarpulli G. (2023). ESKAPE bacteria characterization reveals the presence of *Acinetobacter baumannii* and *Pseudomonas aeruginosa* outbreaks in COVID-19/VAP patients. Am. J. Infect. Control.

[12] Cureño-Díaz M.A., Plascencia-Nieto E.S., Loyola-Cruz M.Á., Cruz-Cruz C., Nolasco-Rojas A.E., Durán-Manuel E.M., Ibáñez-Cervantes G., Gómez-Zamora E., Tamayo-Ordóñez M.C., Tamayo-Ordóñez Y.D.J. (2024). Gram-negative ESKAPE bacteria surveillance in COVID-19 pandemic exposes high-risk sequence types of *Acinetobacter baumannii* MDR in a tertiary care hospital. Pathogens.

[13] Cureño-Díaz M.A., Durán-Manuel E.M., Cruz-Cruz C., Ibáñez-Cervantes G., Rojo-Gutiérrez M.I., Moncayo-Coello C.V., Loyola-Cruz M.Á., Castro-Escarpulli G., Hernández D.M.R.-B., Bello-López J.M. (2021). Impact of the modification of a cleaning and disinfection method of mechanical ventilators of COVID-19 patients and ventilator-associated pneumonia: One year of experience. Am. J. Infect. Control.

[14] Durán-Manuel E.M., Fiscal-Baxin E., Nolasco-Rojas A.E., Loyola-Cruz M.Á., Cruz-Cruz C., Paredes-Mendoza M., López-Ornelas A., Blanco-Hernández D.M.R., Nieto-Velázquez N.G., Rodríguez-Tovar A.V. (2024). Seasonal characterization of the aerobiome in hematopoietic stem cell transplant rooms: Potential risk for immunosuppressed patients. Microorganisms.

[15] Quiroga-Vargas E., Loyola-Cruz M.Á., Rojas-Bernabé A., Moreno-Eutimio M.A., Pastelin-Palacios R., Cruz-Cruz C., Durán-Manuel E.M., Calzada-Mendoza C., Castro-Escarpulli G., Hernández-Hernández G. (2023). Typing of *Candida* spp. from colonized COVID-19 patients reveal virulent genetic backgrounds and clonal dispersion. Pathogens.

[16] Martínez-Ramírez I., Cruz-Cruz C., Ornelas A.L., Duran-Manuel E.M., Estudillo E., Velasco I., Loyola-Cruz M.A., Zayas-Bazán P.G., López-Vargas J.L., Godínez-Cruz Y. (2024). Design, construction and robust validation of a germicidal device based on UV irradiation: A necessity for hospital disinfection in the COVID-19 era. Rev. Mex. Fís..

[17] Beck S.E., Ryu H., Boczek L.A., Cashdollar J.L., Jeanis K.M., Rosenblum J.S., Lawal O.R., Linden K.G. (2017). Evaluating UV-C LED disinfection performance and investigating potential dual-wavelength synergy. Water Res..

[18] El-Bestawy E., Ibrahim M.M., Shalaby E.S.A. (2024). Quantitative and qualitative analysis of bioaerosols emissions from the domestic eastern wastewater treatment plant, Alexandria, Egypt. Sci. Rep..

[19] (2015). Cleanrooms and Associated Controlled Environments—Part 1, Classification of Air Cleanliness by Particle Concentration.

[20] Nicolas-Sayago L., Cruz-Cruz C., Durán-Manuel E.M., Castro-Escarpulli G., Ortíz-López M.G., Jiménez-Zamarripa C.A., Rojas-Bernabé A., Nieto-Velázquez N.G., Tolentino-Sánchez E., Bravata-Alcántara J.C. (2025). Genetic Diversity of *Stenotrophomonas maltophilia* and Clonal Transmission (ST92) in Critical Care Units at Hospital Juárez de México: MLST and Virulence Profiling. Pathogens.

[21] Ji B., Ye W. (2024). Prevention and control of hospital-acquired infections with multidrug-resistant organism: A review. Medicine.

[22] Ng M.K., Mont M.A. (2025). Comparative evaluation of contemporary ultraviolet-C disinfection technologies: UVCeed as a benchmark for smart, portable, and effective pathogen control. GMS Hyg. Infect. Control.

[23] Maugeri A., Casini B., Esposito E., Bracaloni S., Scarpaci M., Patanè F., Milazzo G., Agodi A., Barchitta M. (2025). Impact of ultraviolet light disinfection on reducing hospital-associated infections: A systematic review in healthcare environments. J. Hosp. Infect..

[24] Pfleger S.G., Haertel M.E.M., Plentz P.D.M. (2025). UV-C Disinfection Robots: A Systematic Review. J. Field Rob..

[25] Casini B., Tuvo B., Scarpaci M., Totaro M., Badalucco F., Briani S., Luchini G., Costa A.L., Baggiani A. (2023). Implementation of an Environmental Cleaning Protocol in Hospital Critical Areas Using a UV-C Disinfection Robot. Int. J. Environ. Res. Public Health.

[26] Astrid F., Beata Z., Van den Nest Miriam Julia E., Elisabeth P., Magda D.E. (2021). The use of a UV-C disinfection robot in the routine cleaning process: A field study in an Academic hospital. Antimicrob. Resist. Infect. Control.

[27] Byun J., Byun J., Kang J., Yi I., Lee J., Noh K., Kim J., Choi Y., Chung G., Oh S. (2025). Autonomous Ultraviolet-C Disinfection and Wiping Robot: Assessment in a Hospital Environment.

[28] Jo Freire J.D.O.P., Paes G.O., Gonzalez C.M., Barreiros M.D.G.C., Ferreira A.L.P. (2024). Luz UVC como estratégia de desinfecção do ar e superfícies hospitalares. Acta Paul. Enferm..

[29] Buonanno M., Welch D., Shuryak I., Brenner D.J. (2025). Far-UVC light (222 nm) efficiently and safely inactivates airborne human coronaviruses. Sci. Rep..

[30] Kowalski W. (2010). Ultraviolet Germicidal Irradiation Handbook: UVGI for Air and Surface Disinfection.

[31] Wilson A.P., Smyth D., Moore G., Singleton J., Jackson R., Gant V. (2011). The impact of enhanced cleaning within the intensive care unit on contamination of the near-patient environment with hospital pathogens: A randomized crossover study in critical care units in two hospitals. Crit. Care Med..

[32] Martins Simões P., van der Mee-Marquet N., Youenou B., Ranc A.G., Dupieux-Chabert C., Menard G.S. (2025). *haemolyticus* neonatal infections Study Group. Epidemiology of *Staphylococcus haemolyticus* nosocomial bacteraemia in neonatal intensive care units, France, 2019 to 2023, Predominance of the ST29 (CC3) multidrug-resistant lineage. Euro. Surveill..

[33] Otto M. (2009). *Staphylococcus epidermidis*—The ‘accidental’ pathogen. Nat. Rev. Microbiol..

[34] Jorge R., Teixeira S., Marques M., Pereira J., Paiva J.A. (2024). *Enterococcus hirae* bacteremia associated with traumatic soft tissue infection: A case report. Cureus.

[35] Rollán-Martínez Herrera M., González-Urdiales P., Zubizarreta-Zamalloa A., Rodríguez-Merino E., Martínez-Dubarbie F. (2022). Central nervous system infection by *Bacillus cereus*: A case report and literature review. Rev. Neurol..

[36] Bentur H.N., Dalzell A.M., Riordan F.A.I. (2007). Central venous catheter infection with *Bacillus pumilus* in an immunocompetent child: A case report. Ann. Clin. Microbiol. Antimicrob..

[37] Tsonis I., Karamani L., Xaplanteri P., Kolonitsiou F., Zampakis P., Gatzounis G., Marangos M., Assimakopoulos S.F. (2018). Spontaneous cerebral abscess due to *Bacillus subtilis* in an immunocompetent male patient: A case report and review of literature. World J. Clin. Cases.

[38] Larsen A.L., Pedersen T., Sundsfjord A., Ross T.A., Guleng A.D., Haug J.B., Pöntinen A.K., Samuelsen Ø. (2024). Hospital toilets and drainage systems as a reservoir for a long-term polyclonal outbreak of clinical infections with multidrug-resistant *Klebsiella oxytoca* species complex. Infect. Prev. Pract..

[39] Neog N., Phukan U., Puzari M., Sharma M., Chetia P. (2021). *Klebsiella oxytoca* and emerging nosocomial infections. Curr. Microbiol..

[40] Macesic N., Cottingham H., Wisniewski J.A., Blakeway L.V., Theegala R., Pragastis K., Stewardson A., Bass P., Gritt M., Spilsbury S. (2025). Hospital *Enterococcus faecium* demonstrates distinct environmental and patient reservoirs: A genomic point prevalence survey. Infect. Control Hosp. Epidemiol..

[41] Moreno-Torres D., Jiménez-Zamarripa C.A., Munguía-Mogo S.M., Calzada-Mendoza C.C., Cruz-Cruz C., Durán-Manuel E.M., Gutiérrez-Ramírez A., Castro-Escarpulli G., Vélez-Cruz M.E., Sosa-Hernández O. (2025). Comprehensive assessment of health risks associated with Gram-negative bacterial contamination on healthcare personnel gowns in clinical settings. Microorganisms.

[42] Harper H., Logan J., Kubat R., Jones M. (2022). *Leclercia adecarboxylata* catheter-related bacteraemia in an immunocompromised patient. BMJ Case Rep..

[43] Rutala W.A., Weber D.J. (2019). Best practices for disinfection of noncritical environmental surfaces and equipment in health care facilities: A bundle approach. Am. J. Infect. Control.

[44] Binns R., Li W., Wu C.D., Campbell S., Knoernschild K., Yang B. (2020). Effect of ultraviolet radiation on *Candida albicans* biofilm on poly(methylmethacrylate) resin. J. Prosthodont..

[45] Różańska A., Walkowicz M., Bulanda M., Kasperski T., Synowiec E., Osuch P., Chmielarczyk A. (2023). Evaluation of the efficacy of UV-C radiation in eliminating microorganisms of special epidemiological importance from touch surfaces under laboratory conditions and in the hospital environment. Healthcare.

[46] Hussaini I.M., Oyewole O.A., Sulaiman M.A., Dabban A.I., Sulaiman A.N., Tarek R. (2024). Microbial anti-biofilms: Types and mechanism of action. Res. Microbiol..

[47] Khan M., McDonald M., Mundada K., Willcox M. (2022). Efficacy of ultraviolet radiations against coronavirus, bacteria, fungi, fungal spores and biofilm. Hygiene.

[48] Bello-López J.M. (2026). Impact of a UV-C multiemitter disinfection system on hospital environmental bioburden and inactivation of clinically relevant pathogens. Mendeley Data.

